# Prediction of Social Engagement in Long-Term Care Homes by Sex: A Population-Based Analysis Using Machine Learning

**DOI:** 10.1177/07334648241290589

**Published:** 2024-10-12

**Authors:** Ali Abedi, Shehroz S. Khan, Andrea Iaboni, Susan E. Bronskill, Jennifer Bethell

**Affiliations:** 1 KITE Research Institute, Toronto Rehabilitation Institute, 7989 University Health Network, Toronto, ON, Canada; 2 ICES, Toronto, ON, Canada; 3 Department of Psychiatry, Temerty Faculty of Medicine, University of Toronto, Toronto, ON, Canada; 4 Institute of Health Policy, Management and Evaluation, University of Toronto, Toronto, ON, Canada; 5 Sunnybrook Research Institute, Sunnybrook Health Sciences Centre, Toronto, ON, Canada

**Keywords:** nursing homes, social participation, machine learning, female, male

## Abstract

The objective of this study was to use population-based clinical assessment data to build and evaluate machine-learning models for predicting social engagement among female and male residents of long-term care (LTC) homes. Routine clinical assessments from 203,970 unique residents in 647 LTC homes in Ontario, Canada, collected between April 1, 2010, and March 31, 2020, were used to build predictive models for the Index of Social Engagement (ISE) using a data-driven machine-learning approach. General and sex-specific models were built to predict the ISE. The models showed a moderate prediction ability, with random forest emerging as the optimal model. Mean absolute errors were 0.71 and 0.73 in females and males, respectively, using general models and 0.69 and 0.73 using sex-specific models. Variables most highly correlated with the ISE, including activity pursuits, cognition, and physical health and functioning, differed little by sex. Factors associated with social engagement were similar in female and male residents.

**What this paper adds**

• Predictive models using a data-driven machine learning approach can moderately predict social engagement among long-term care home residents.
• The predictor variables that correlate with social engagement are consistent across both female and male residents.
• The performance of general models, developed using the data from both female and male residents, and sex-specific models, developed using the data collected through female or male residents only, is similar.

**Applications of study findings**

• Sex may have a relatively minor role in tailoring approaches to addressing social engagement for residents of long-term care homes.
• The developed predictive models can be run on routinely collected data from residents, enabling targeted interventions to enhance social engagement in long-term care homes.
• The lack of sex differences suggests that the impacts of gender (i.e., socially constructed roles, behaviors, activities, and attributes) may be flattened by the widespread impacts of disability with loss of independence and social network, coupled with the pervasive effects of ageism and ableism.

## Introduction

Social engagement, as an objective aspect of social connection, pertains to taking part in real-life activities within the communities in which individuals live and can include leisure or productive activities (Glass et al., 2006). Social engagement has been highlighted as a key concept for research on social connection in long-term care (LTC) homes (Leedahl et al., 2018), also known as residential care or nursing homes, and continuing care facilities. Social engagement among older adults residing in LTC homes is inversely associated with mortality (Pastor-Barriuso et al., 2020), cognitive decline (Freeman et al., 2017), responsive behavior (Choi et al., 2018), and depression (Lou et al., 2013), and is positively associated with better mental (Bethell et al., 2021) and physical (Lem et al., 2021) health. Early in the COVID-19 pandemic, lack of contact with family or friends was associated with increased mortality among residents of LTC homes, thought to be related to reduced social support and increased social isolation and loneliness (Savage et al., 2022).

Although some studies have reported that the level of social engagement is similar across female and male residents in LTC homes (Achterberg et al., 2003; Bliss et al., 2017; Kang, 2012), there is also some evidence that males may be more socially isolated when considering personal contact with family or friends outside the LTC home (Chamberlain et al., 2020). Among older adults more broadly, some research has suggested that males are more socially isolated than females (Menec et al., 2019), whereas others have shown the opposite but with the nuance that this difference may be modified by other factors (Cloutier-Fisher & Kobayashi, 2009; Umberson et al., 2022). A review has outlined potential risk factors for social isolation among LTC home residents, encompassing factors at the individual, home, and societal levels (Boamah et al., 2021). In particular, resident-level factors may pose specific challenges to social engagement, including dementia and cognitive impairment (Moyle et al., 2011), sensory (i.e., vision and/or hearing) impairment (Guthrie et al., 2018), and communication difficulties (Resnick et al., 1997). At the level of the LTC home, factors such as care philosophy, size, staffing, location, and community characteristics (Clemens et al., 2022) have all been investigated for their impact on aspects of social connection. More broadly, the negative impacts of structural determinants of health, such as ageism and ableism, continue to exclude LTC homes and their residents (Herron et al., 2021).

However, little is known about potential sex or gender differences in the predictors of social connection among residents of LTC homes. In their study, Umberson et al. apply two theoretical perspectives of gender to examine how gender differences in social isolation change over the life course (Umberson et al., 2022); first, that gender differences in individual decision-making are a function of the opportunities that are available to men and women and the structures that control these opportunities and, second, that social contexts such as marriage have gendered effects (Springer et al., 2012). In short, gendered influences, expectations, and constraints have direct implications for social connection (Umberson et al., 2022). Aside from gendered influences on social connection for older adults more generally, gender may also have specific considerations for LTC homes. For example, most of those living, working, and visiting LTC homes are female; nearly two-thirds of residents (Ng et al., 2020), 90% of personal support workers/care aides, the largest group of care providers in LTC homes (Song et al., 2020), and the majority of family caregivers are female.

The objective of this study was to build and evaluate machine-learning models for predicting social engagement among residents of LTC homes, using population-based clinical assessment data, in order to explore potential gender differences. Machine-learning models were used as a data-driven approach to simultaneously analyze a large dataset with numerous resident-level and LTC home-level predictors and multiple types of data. To our knowledge, no previous studies have used population-based data to examine sex or gender differences in factors associated with social engagement among residents of LTC homes. The findings will be useful for hypothesis generation, by identifying potential differences in social engagement among people living in LTC homes and highlighting areas for future research to understand causal effects.

## Methods

### Study Design and Setting

This cross-sectional study was a secondary analysis of population-based routine clinical assessment data from residents of LTC homes in Ontario, Canada. In Canada, LTC homes provide 24-h care 7 days a week, including professional health services and personal care, to residents who typically have complex healthcare needs. LTC homes in Canada are funded or subsidized by provincial or territorial governments (Canadian Institute for Health Information, 2021). Data from the Continuing Care Reporting System (CCRS) were used to identify all residents of LTC homes in the province between April 1, 2010, and March 31, 2020, and obtain their demographic and clinical assessment data. The CCRS is designed to capture information on all residents of all publicly funded continuing care facilities (including LTC homes) in Canada that have 24-h nursing available. LTC homes collect information on the residents they serve (Canadian Institute for Health Information, 2020), using the Resident Assessment Instrument Minimum Data Set (RAI-MDS) (Canadian Institute for Health Information, 2010) which is gathered electronically by trained health professionals at the point of care. CCRS data are aggregated for multiple applications, including care planning, funding, research, quality improvement, and accountability purposes (Hirdes et al., 2013). The CCRS is compiled by the Canadian Institute for Health Information.

These datasets were linked using unique encoded identifiers and analyzed at ICES. ICES is an independent, non-profit research institute funded by an annual grant from the Ontario Ministry of Health (MOH) and the Ministry of Long-Term Care (MLTC). As a prescribed entity under Ontario’s privacy legislation, ICES is authorized to collect and use healthcare data for the purposes of health system analysis, evaluation, and decision support. Secure access to these data is governed by policies and procedures that are approved by the Information and Privacy Commissioner of Ontario.

ICES is a prescribed entity under Ontario’s Personal Health Information Protection Act (PHIPA). Section 45 of PHIPA authorizes ICES to collect personal health information, without consent, for the purpose of analysis or compiling statistical information with respect to the management of, evaluation or monitoring of, the allocation of resources to or planning for all or part of the health system. Projects that use data collected by ICES under section 45 of PHIPA, and use no other data, are exempt from REB review. The use of the data in this project is authorized under section 45 and approved by ICES’ Privacy and Legal Office.

### Study Population

Individuals aged 18 to 110 years who had at least one full annual assessment completed during the study period were eligible for the study. Assessments whereby the resident was identified as potentially short stay (discharge projected within 90 days or uncertain discharge status), comatose, or missing Index of Social Engagement (ISE) were excluded. To account for aggregation in the data (i.e., non-independence of multiple assessment records per individual) and examine length of stay as a potential predictor, one assessment per resident during the study period was randomly selected for analysis.

### Outcome: Index of Social Engagement

The ISE is calculated as the sum of six dichotomous items: (1) at ease interacting with others; (2) at ease doing planned or structured activities; (3) at ease doing self-initiated activities; (4) establishes own goals; (5) pursues involvement in the life of the LTC home; and (6) accepts invitations into most group activities (Mor et al., 1995). ISE values range from 0 to 6, where higher scores indicate a higher level of social engagement. For the analysis, the ISE was treated as a seven-level ordinal variable.

### Predictors: Resident- and Home-Level Variables

Each RAI-MDS assessment captures a resident’s demographic information as well as measures of their cognition, communication, hearing, vision, mood and behavior, psychosocial well-being, physical functioning and structural problems, continence, disease diagnoses, health conditions, nutritional status, dental status, skin condition, activity pursuits, special treatments and procedures, discharge potential, and medications. In addition to the ISE, other RAI-MDS scales are calculated from these data, including measures of depression, pain, activities of daily living, aggressive behavior, pressure ulcer risk, cognitive performance, and changes in health (CIHR, 2013; Mor et al., 1995). LTC home-level variables are also available including the number of beds. Urban/rural status and neighborhood income quintiles were derived through linking Canadian census data to the LTC home postal code (PCCF, 2013). All variables were considered for the analysis except those directly related to the ISE outcome; the six individual ISE items and two additional variables calculated using ISE items (i.e., Social Relationship Clinical Assessment Protocol [CAP] and Activities CAP) (Canadian Institute for Health Information, 2008) were excluded.

### Sex-Stratified Analysis

While potential differences in predictors of social engagement were theorized in this study as relating to socially constructed roles, behaviors, activities, and attributes that a given society considers appropriate for men and women (i.e., gender) (WHO, 2022), the data available for this study were limited to biological sex. Therefore, to explore the potential influence of gender, study analyses were conducted and reported disaggregated by sex (Morgan et al., 2016). That is, machine-learning methods were used to develop and evaluate general and sex-specific models in order to compare their performance.

### Statistical Analysis and Predictive Modeling

Descriptive statistics (means and proportions) were used to describe the study sample overall and by sex. To compare the mean ISE between females and males, within variable subcategories, *p*-values were calculated using independent samples t-test. A data-driven machine-learning pipeline was then applied to build predictive models for the ISE; in this approach, variables were selected based entirely on statistical rather than clinical reasoning. The analysis consisted of four steps:

### Step 1: Preprocessing

Univariate statistics, including missing values, were examined for all variables to ensure they would contribute informative data to the predictive models. Variables were excluded if they were (1) unique identifiers (e.g., health record numbers); (2) zero-variance (e.g., after excluding comatose residents, all remaining assessments had identical values for this variable); (3) near-zero variance (i.e., variables with variance less than 0.01) (Kuhn & Johnson, 2013); and (4) missing more than 30% of values (Khan et al., 2019). For numerical and categorical variables with less than 30% missing values, mean, and mode missing value imputation (Khan et al., 2019) was performed, respectively. The final set of variables includes categorical, dichotomous, ordinal, interval, and ratio variables, making it a mixed dataset. All variables were entered into the analysis as originally coded or re-coded in the case of missing values (as described above).

### Step 2: Variable Selection

Variable selection is the process of picking out the most relevant variables for building predictive models. Machine-learning models are prone to be misled by irrelevant input variables, resulting in poorer prediction performance (Chen et al., 2020; Li et al., 2018). The correlation-based feature selection approach (Li et al., 2018) was used in this study in the full sample. First, the correlations between all predictor variables and the outcome variable (ISE) were calculated, and the predictor variables whose absolute value correlation with the ISE was greater than 0.10 were retained. The correlations between all predictor variables were also calculated, and among predictor variables correlated with each other, that is, > 0.75, the one with the highest correlation with the ISE was retained. To calculate the correlation between predictors, and between predictors and the outcome variable (ISE), various correlation measures were employed because of the diverse types of variables in the dataset. For instance, rank biserial, correlation ratio, and Kendall rank correlation coefficient were used to calculate the correlation between an ordinal variable and dichotomous, categorical, and ordinal variables, respectively (Khamis, 2008). These steps were conducted overall as well as separately for females and males, allowing the possibility of retaining distinct sets of variables.

### Step 3: Prediction

Five types of predictive machine-learning models were implemented in the full sample: (1) Logistic Regression (LR), (2) Random Forest (RF), (3) Support Vector Machine (SVM) with linear kernel, (4) SVM with Radial Basis Function (RBF) kernel, and (5) 4-layer fully connected Neural Network (NN). Since the ISE is an ordinal variable, the ordinal version (Cardoso & Sousa, 2011; Kook et al., 2022) of the above models was implemented. The nested five-fold Cross-Validation (CV) approach (Spasojevic et al., 2021) was implemented for training, tuning hyperparameters, and evaluating the models.

Different encoding approaches were used to prepare the variables to be input into the predictive models as the dataset is mixed. The interval and ratio variables remained unchanged, the categorical and dichotomous variables were one-hot encoded, and the ordinal variables were ordinally encoded.

Four metrics were used to evaluate the performance of the predictive models by comparing each individual’s actual ISE with the value predicted by the model: (1) accuracy, the number of correct predictions divided by the total number of samples; (2) Ordinal Classification Index (OCI) (Cardoso & Sousa, 2011); (3) Mean Absolute Error (MAE); and (4) R2 score (coefficient of determination). For accuracy and R2, higher scores indicate better model performance, whereas for OCI and MAE lower scores indicate better model performance.

The best machine-learning model, based on a comparison of the evaluation metrics, was used in the subsequent analysis whereby six different five-fold CV settings were implemented to explore sex differences. Two cross-sex models (male–female and female–male) built the model on one sex and evaluated it on the other. Two sex-specific models (male–male and female–female) built and evaluated the models separately by sex. The two remaining models (both sexes-male and both sexes-female) built the model on the full sample and then evaluated separately by sex. The same evaluation metrics were used to compare the performance of these models.

### Step 4: Variable Importance Ranking

In order to enhance the explainability of the developed predictive machine-learning models, an analysis of variable/feature importance ranking (Chen et al., 2020; Li et al., 2018) was performed on the best-performing sex-specific models. In this ranking, features were evaluated and ranked according to their contribution to the prediction by the model, considering their interactions with each other.

## Results

**Figure 1.**
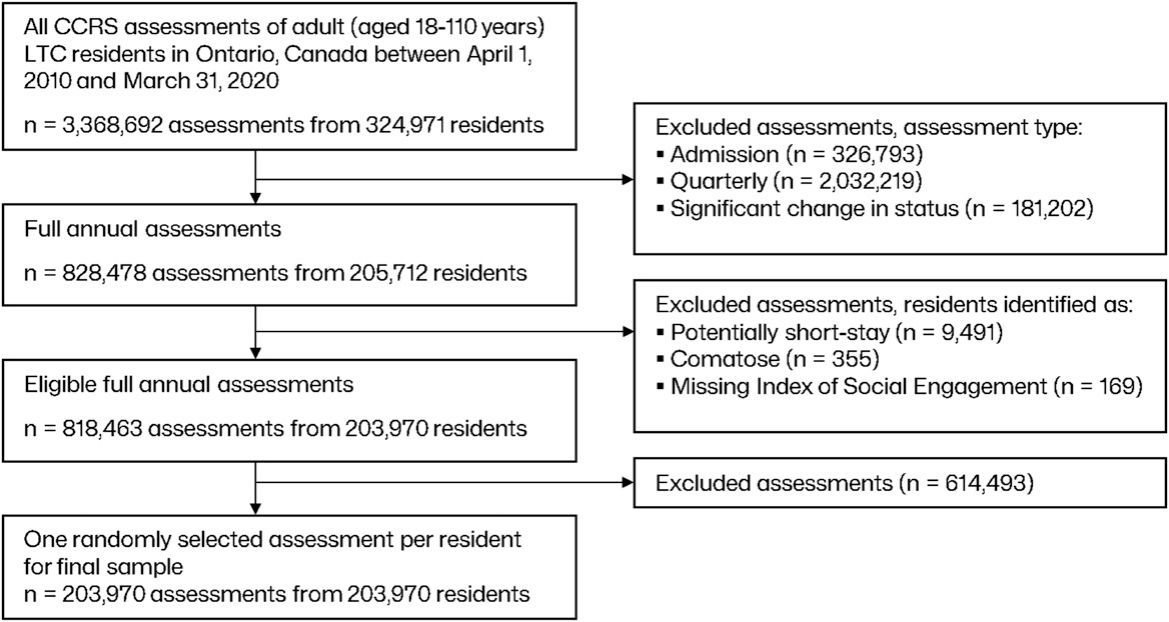
Cohort selection hierarchy.

Over the 10-year study period (2010–2020), there were 3,368,692 assessments from 324,971 unique individuals. After exclusions, there were 818,463 full annual assessments from 203,970 unique residents, and after randomly selecting one assessment per resident, the final sample consisted of 203,970 full annual assessments from 203,970 unique residents (see Figure 1).

Table 1 provides selected demographic, clinical, and home-level characteristics, with the mean and standard deviation of the ISE across different subcategories of these characteristics, stratified by sex. The study sample consisted of 203,970 residents of LTC homes, of which just over two-thirds (68.4%) were female. Among females, roughly two-thirds were aged 85 years and older (64.2%) and most were widowed (69.5%). Among males, less than half were aged 85 years and older (45.8%) and 44.0% were married. For both females and males, roughly half of the residents had been living in the LTC home for over 1 year (56.5% and 48.3%, respectively) and most residents spoke English as their primary language (80.1% and 80.5%, respectively) and had less than high school education (58.5% and 56.6%, respectively). The mean (standard deviation) of the ISE among females and males was 2.98 (1.76) and 2.93 (1.68), respectively. In many variable subcategories, females had a higher mean ISE than males. However, in certain subcategories, there was no difference, such as among those severely impaired in daily activities (mean ISE: 2.18 in males and 2.18 in females), or males demonstrated a higher mean ISE, such as among married residents (mean ISE: 2.76 in males and 2.72 in females).

**Table 1.** Proportion of Residents and the Index of Social Engagement (ISE), Stratified by Sex, by (a) Resident and (b) Long-Term Care (LTC) Home-Level Variables, in Assessments Collected From 203,970 Residents Between April 1, 2010, and March 31, 2020. The *p*-Values, Calculated Using Independent Samples t-test, Compare the Mean ISE Between Females and Males Within Subcategories.

	Proportion of Residents^a^		ISE Mean (SD)		*p*-Value
	Females	Males	Females	Males	
(a) Resident-level variables					
Sex					
Female	100.0	0.0	2.98 (1.76)		0.6942
Male	0.0	100.0		2.93 (1.68)	0.6942
Age category (years)					
18–64	3.5	7.6	3.31 (1.84)	3.23 (1.72)	0.7560
65–74	7.0	13.5	3.14 (1.84)	2.98 (1.70)	0.0615
75–84	25.3	33.1	2.97 (1.80)	2.83 (1.69)	<0.0001
≥85	64.2	45.8	2.94 (1.73)	2.93 (1.65)	0.0177
Length of stay (months)					
≤12	43.5	51.7	3.17 (1.71)	3.00 (1.64)	<0.0001
13–48	41.1	38.0	2.98 (1.75)	2.90 (1.69)	0.0221
>48	15.4	10.3	2.43 (1.83)	2.69 (1.78)	<0.0001
Marital status					
Never married	6.8	12.4	3.13 (1.79)	3.14 (1.70)	0.0465
Married	16.4	44.0	2.72 (1.77)	2.76 (1.68)	<0.0001
Widowed	69.5	32.1	3.00 (1.75)	3.02 (1.66)	<0.0001
Separated	1.6	3.5	3.16 (1.79)	3.08 (1.63)	0.7239
Divorced	5.7	8.0	3.20 (1.74)	3.08 (1.63)	0.0410
Primary language category					
English	80.1	80.5	3.05 (1.76)	2.99 (1.67)	0.4213
French	3.7	3.5	3.02 (1.86)	2.98 (1.80)	0.8407
Other	16.2	16.0	2.63 (1.69)	2.64 (1.64)	0.5180
Highest level of education					
8th Grade or less	58.5	56.6	2.93 (1.77)	2.89 (1.68)	0.4254
At least some high school	27.0	23.0	3.06 (1.76)	3.01 (1.67)	0.6328
Technical/trade school or college	9.3	11.2	3.06 (1.75)	2.98 (1.68)	0.0034
Bachelor’s or graduate degree	5.2	9.2	2.97 (1.74)	2.90 (1.66)	0.3718
Cognitive performance scale					
0–2	32.9	34.8	3.93 (1.53)	3.73 (1.49)	<0.0001
3	35.6	35.8	3.08 (1.62)	2.98 (1.56)	<0.0001
4–6 (Most impaired)	31.5	29.4	1.86 (1.51)	1.93 (1.49)	0.0003
Activities of Daily Living Short Form Scale					
0–5	16.3	19.7	4.07 (1.54)	3.79 (1.50)	<0.0001
6–10	39.0	40.0	3.43 (1.61)	3.26 (1.57)	<0.0001
11–16 (Most impaired)	44.7	40.3	2.18 (1.61)	2.18 (1.55)	0.9878
Aggressive Behavior Scale					
0	54.5	49.2	3.35 (1.73)	3.30 (1.62)	0.0451
1–4	35.8	39.6	2.66 (1.70)	2.69 (1.65)	0.0012
5–12 (Highest levels)	9.7	11.2	2.04 (1.61)	2.13 (1.59)	0.0081
Hearing					
Hears adequately	60.8	59.7	3.08 (1.77)	3.02 (1.68)	0.9411
Minimal difficulty	25.0	25.8	2.94 (1.75)	2.91 (1.68)	0.9486
Hears in special situations only	12.4	12.8	2.67 (1.70)	2.61 (1.62)	0.5137
Highly impaired	1.8	1.7	2.20 (1.73)	2.29 (1.64)	0.4918
Vision					
Adequate	54.3	57.2	3.30 (1.72)	3.17 (1.64)	<0.0001
Impaired	26.7	26.6	2.94 (1.71)	2.88 (1.64)	0.4175
Moderately to severely impaired	19.0	16.2	2.12 (1.67)	2.10 (1.63)	0.3460
Number of medications (per week)					
<5	21.6	20.9	2.64 (1.77)	2.73 (1.70)	<0.0001
5–10	31.8	31.3	2.81 (1.75)	2.80 (1.67)	0.0675
11–15	32.9	34.2	3.13 (1.74)	3.02 (1.67)	<0.0001
>15	13.7	13.6	3.52 (1.68)	3.29 (1.63)	<0.0001
Average time involved in activities					
Most—More than 2/3 of time	19.0	16.9	4.43 (1.50)	4.30 (1.48)	0.0012
Some—From 1/3 to 2/3 of time	57.2	57.2	3.02 (1.57)	3.01 (1.51)	0.0031
Little—Less than 1/3 of time	22.4	24.1	1.76 (1.44)	1.89 (1.42)	<0.0001
None	1.4	1.8	1.17 (1.31)	1.35 (1.32)	0.0069
Long-term memory					
Memory OK	33.1	37.4	3.89 (1.54)	3.68 (1.50)	<0.0001
Memory problem	66.9	62.6	2.52 (1.69)	2.48 (1.62)	0.0099
Resident lived alone prior to admission					
Did not live alone	84.7	88.2	2.93 (1.76)	2.89 (1.68)	0.9064
Lived alone	15.3	11.8	3.25 (1.78)	3.25 (1.65)	0.0122
(b) LTC home-level variables					
LTC home urban/rural status					
Urban	87.3	86.3	2.96 (1.75)	2.92 (1.66)	0.4863
Rural	12.7	13.7	3.13 (1.86)	3.01 (1.76)	0.0017
LTC home number of beds					
0–48	2.5	2.4	3.26 (1.96)	3.07 (1.85)	0.1663
49–96	18.2	18.2	3.03 (1.84)	2.94 (1.74)	0.0270
97–300	73.0	72.6	2.98 (1.75)	2.95 (1.66)	0.0673
301–480	6.3	6.8	2.72 (1.61)	2.62 (1.58)	0.0120
LTC home neighborhood income quintile					
Lowest income quintile	32.4	33.3	2.95 (1.75)	2.87 (1.69)	0.0252
2nd income quintile	20.0	19.8	2.94 (1.76)	2.93 (1.67)	0.0412
3rd income quintile	16.6	17.7	2.99 (1.82)	2.96 (1.71)	0.3280
4th income quintile	17.5	16.6	3.04 (1.75)	3.01 (1.66)	0.3028
Highest income quintile	13.5	12.6	2.99 (1.74)	2.92 (1.65)	0.0333

^a^proportion (%) of female and male residents in each variable subcategory.

**Figure 2.**
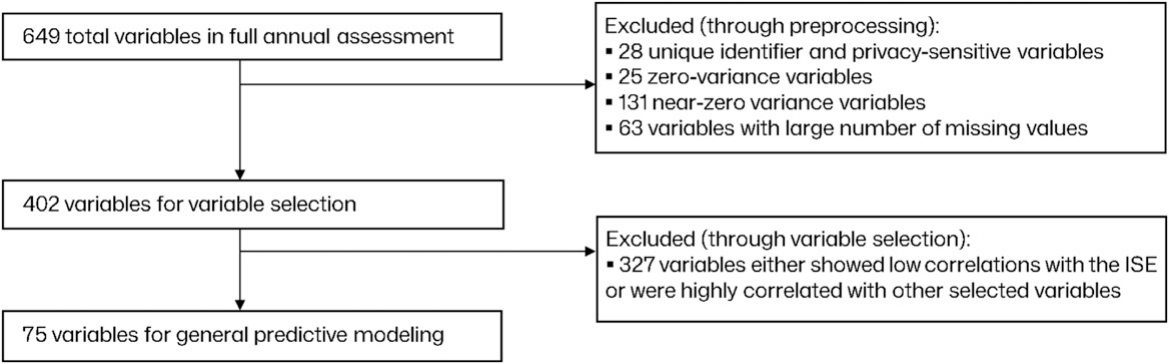
Preprocessing and variable selection for the general model.

The initial data consisted of 649 variables. In the preprocessing step, 402 variables were retained and considered for variable selection (see Figure 2). Table 2 lists the highest absolute correlation variables (i.e., > 0.30) selected through the correlation-based feature selection, separately for females and males. Variables with positive-sign correlation values reflect attributes such as lesser impairment, greater independence, and more activity pursuit, and vice versa.

**Table 2.** Correlation of Variables (Chosen Through the Correlation-Based Feature Selection) With the Index of Social Engagement (ISE) in Assessments Collected From 203,970 Residents in LTC Between April 1, 2010, and March 31, 2020, Stratified by Sex. Only Variables With a Correlation Greater Than 0.30 Among Females are Shown.

		Females		Males	
Variable Name^a^	Variable Category^a^	Correlation^b^	Rank	Correlation^b^	Rank^c^
Average Time Involved in Activities	Activity Pursuit Patterns	−0.50^d^	1	−0.47	1
Cognitive Performance Scale	Outcome Scale	−0.49	2	−0.45	2
Independent Eating	Physical Functioning and Structural Problems	−0.47	3	−0.40	3
Able to Locate Own Room	Cognitive Patterns	0.43	4	0.39	5
Prefer Playing Cards and Other Games	Activity Pursuit Patterns	0.41	5	0.36	8
Able to Determine that is in a Facility	Cognitive Patterns	0.41	6	0.38	7
Able to Identify the Current Season	Cognitive Patterns	0.41	7	0.38	6
Bowel Incontinence	Continence	−0.40	8	−0.36	9
Prefer Trips or Shopping	Activity Pursuit Patterns	0.38	9	0.35	11
Prefer Talking or Conversing	Activity Pursuit Patterns	0.37	10	0.35	10
Prefer Playing Crafts/Arts	Activity Pursuit Patterns	0.37	11	0.24	28
Able to Distinguish Staff Names/Faces	Cognitive Patterns	0.37	12	0.35	13
Prefer Helping Others	Activity Pursuit Patterns	0.37	13	0.33	15
Strong Identification with Past Roles and Life Status	Psychosocial Well-Being	0.37	14	0.35	12
Balance Problem While Sitting	Physical Functioning and Structural Problems	−0.37	15	−0.32	17
Long-Term Memory Problem	Cognitive Patterns	−0.37	16	−0.35	14
Prefer Reading/Writing	Activity Pursuit Patterns	0.36	17	0.32	18
Prefer Exercise/Sports	Activity Pursuit Patterns	0.36	18	0.32	19
Speech Clarity	Communication/Hearing Patterns	−0.34	19	−0.29	22
Outside Facility Activity Preference	Activity Pursuit Patterns	0.33	20	0.32	16
Mechanically Altered Diet	Oral/Nutritional Status	−0.33	21	−0.27	25
Bladder Incontinence	Continence	−0.33	22	−0.30	21
Pressure Ulcer Risk Scale	Outcome Scale	−0.31	23	−0.28	23
Inside Facility/Off Unit Activity Preference	Activity Pursuit Patterns	0.31	24	0.31	20

^a^The variable names and categories were taken from the RAI-MDS guidelines for full annual assessments (Canadian Institute for Health Information, 2010).

^b^The *p*-values for all calculated correlations were statistically significant at *p* < .00001.

^c^The 4th and 24th ranked variables in males are “Ability to Understand Others,” categorized under “Communication/Hearing Patterns” with an ISE correlation of −0.40, and “Short-Term Memory Problem,” categorized under “Cognitive Patterns” with an ISE correlation of −0.27. These two variables were excluded for females during correlation-based feature selection because they were highly correlated with selected variables.

^d^The sign of the correlation is contingent on the variable’s coding (Canadian Institute for Health Information, 2010); higher scores on variables do not necessarily indicate higher functioning or more involvement in activities, for example, for “Average Time Involved in Activities,” lower values indicate a greater amount of time being spent in activities, whereas for some other activity pursuit variables, higher values indicate taking part in specific activities.

In the variable selection step, there were some apparent sex differences; the total number of variables retained 75 (overall), 77 (for females), and 75 (for males) was similar. The correlation-based ranking of variables was also similar by sex. For example, in Table 2, 9 of the 10 highest-ranked variables in males were also among the 10 highest-ranked variables in females. However, the absolute correlation values were generally lower in males than in females. The list of variables used for the prediction of the ISE is provided in Table S1 in Supplemental Materials.

Table 3 shows that, of the five predictive modeling approaches, the ordinal RF model achieved the best performance with the highest accuracy (0.43) and R2 score (0.65) as well as the lowest MAE (0.72) and OCI (0.63). Table 4 presents the performance of the ordinal RF model in predicting the ISE in the six different distributions of samples in the dataset used for building and evaluating predictive models. In predicting ISE among either males or females, cross-sex models (male–female and female–male) performed poorly across evaluation metrics. The performance of the sex-specific models (male–male and female–female) was relatively better than that of other models, with the exception of the OCI, which favored models based on both sexes. Yet, overall, evaluation metrics indicated that sex-specific modeling approaches had little impact on model performance. ISE prediction was slightly better among females.

**Table 3.** Model Performance in Predicting the ISE for the Entire (Both Sexes) Dataset Based on Model Type and Evaluation Metrics: Accuracy, Ordinal Classification Index (OCI), Mean Absolute Error (MAE), and R2 Score. The Bolded Values Indicate the Best Results.

Ordinal Predictive Model	Evaluation Metric			
	Accuracy	OCI	MAE	R2
Logistic regression	0.42	0.64	0.78	0.57
Random forest	**0.43**	**0.63**	**0.72**	**0.65**
Support vector machine with linear kernel	0.40	0.66	0.79	0.61
Support vector machine with RBF kernel	0.40	0.65	0.78	0.61
Neural network	0.41	0.69	0.79	0.57

**Table 4.** Ordinal Random Forest Model Performance in Predicting the ISE by Sex and Based on Evaluation Metrics: Accuracy, Ordinal Classification Index (OCI), Mean Absolute Error (MAE), and R2 Score. The Bolded Values Indicate the Best Results According to Sex in Which the Model was Evaluated.

Sex Distribution of the Datasets Used for Model Building and Evaluation		Evaluation Metric			
		Accuracy	OCI	MAE	R2
Model Built On	Model Evaluated On				
Both sexes	Females	0.44	**0.61**	0.71	0.64
Females	Females	**0.45**	0.62	**0.69**	**0.66**
Males	Females	0.43	0.67	0.72	0.66
Both sexes	Males	0.43	**0.64**	0.73	0.61
Males	Males	**0.44**	0.65	**0.73**	**0.62**
Females	Males	0.42	0.66	0.74	0.58

**Figure 3.**
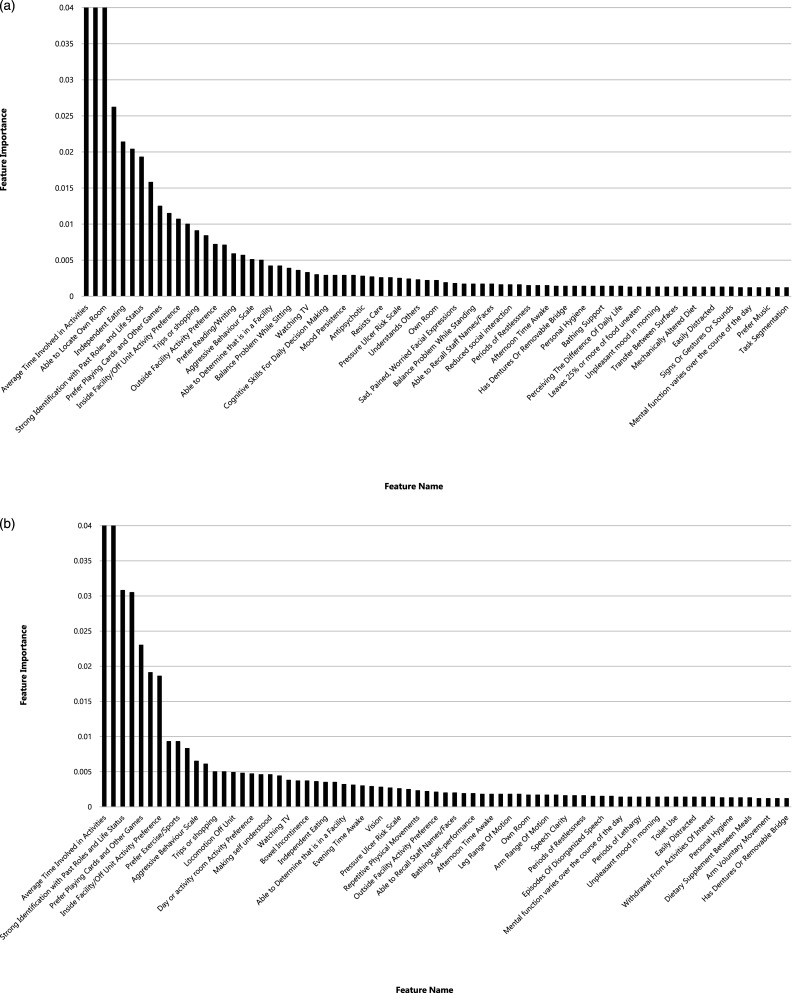
Feature importance ranking for the ordinal random forest model predicting the Index of Social Engagement (ISE) among (a) female and (b) male residents. For detailed information on the features (predictor variables), please refer to supplemental material Table S1.

The feature importance rankings for all variables in the sex-specific ordinal RF models are presented in Figure 3. Of the retained variables, 68 were common to both models, whereas nine were selected only for the female-specific model and seven were selected only for the male-specific model. However, the variables retained in a single sex-specific model were of low importance. Most of the variables with the largest effects on their respective models were common to both models, related to activity pursuit patterns (i.e., time in activities and activity preference for helping others, playing card or other games, talking or conversing, and preference for activities inside the facility but off unit), cognitive patterns (i.e., able to locate own room), and well-being (i.e., strong identification with past roles and life status). Additional variables were also important in the female-specific model, similarly related to cognitive patterns (i.e., cognitive performance scale, memory/recall of current season, and long-term memory problems) and activity pursuit patterns (i.e., activity preference for exercise/sport); with the exception of memory/recall of current season, these variables were also among highly ranked variables in the male-specific model. The independent eating variable was of high importance only in the female-specific model, although its correlation with the ISE was similar for both females and males (Table 2). The feature importance ranking from the ordinal RF model further accounts for the interaction of variables with each other in making predictions, reflecting how the model operates in real-world applications.

## Discussion

Utilizing a data-driven machine-learning pipeline approach, general and sex-specific models were developed for predicting social engagement among residents of LTC homes. The models exhibited moderate performance, with the RF outperforming the other evaluated models. The variables included in predictive modeling were those related to activity pursuits, cognition, and physical health and functioning and were similar in females and males. The lack of sex differences in these variables, coupled with the relatively similar performance between the general and sex-specific models, implies that sex has a relatively minor impact on how the variables predict social engagement among residents in LTC homes.

Few studies have addressed potential sex or gender differences in the predictors of social connection among older adults. However, evidence suggests potential differences. For example, a recent analysis using data from a large survey in Germany found that the impact of disability on loneliness was stronger for females than males (Pagan, 2020). Similarly, analysis of a national survey in Sweden showed the predictors of loneliness differed somewhat between females and males such that mobility problems and mobility reduction were predictors only among females, whereas low levels of social contact and social contact reduction predicted loneliness only among males (Dahlberg et al., 2015). While less is known about potential sex or gender differences in the predictors of social connection among residents of LTC homes, the apparent lack of sex differences observed in this study is corroborated by a recent scoping review of social isolation among older adults in LTC; the review reported risk factors at multiple levels, however, did not report findings related to sex or gender as a predictor or modifier of these factors (Boamah et al., 2021).

RFs handle large datasets efficiently through parallel processing of multiple decision trees, making them ideal for population-based studies. They capture complex variable interactions by exploring different data subsets, uncovering relationships that simpler models might miss. RFs accommodate various data types, categorical, ordinal, interval, and ratio, making them versatile for diverse datasets. In this study, the ordinal RF model is particularly suited to predicting outcomes such as ISE. RFs also offer built-in feature importance ranking, enhancing explainability and helping identify key predictors, providing valuable insights for researchers and policymakers to translate findings into actionable policies. The findings from the feature importance rankings, unsurprisingly, highlight variables related to cognition for both men and women; symptoms of dementia, compounded by others’ lack of understanding about dementia, can negatively impact social engagement (Moyle et al., 2011). Notably, strong identification with past roles and life status was also important in both models, which aligns with the body of research on reminiscence therapy, whereby individuals recall past events, activities, and experiences, and has been studied for its impact on social connection (Mikkelsen et al., 2019). Other important variables related to activities in LTC homes (e.g., playing cards or games, exercise, and sport) were identified but also pointed to the importance of residents’ experience of giving social support; indeed, others have proposed social productivity and peer support as pillars of the social revolution that is needed in residential care (Theurer et al., 2015). Independent eating, which was important in the female-specific model, highlights the social aspects of mealtimes in LTC homes (Keller et al., 2014) and suggests a potentially gendered effect that could relate to roles and expectations when eating with others (Rolls et al., 1991).

The results of this study hold significance for research as well as planning and providing care in LTC homes. For the latter, the potential implications for policy and practice are twofold. First, the findings suggest that sex may have a relatively minor role in tailoring approaches to addressing social engagement for residents of LTC homes. Second, the study introduces the potential to leverage predictive models developed and run on routinely collected data, enabling targeted interventions to enhance social engagement in LTC homes. In particular, those conducting clinical assessments might use these data to identify residents with low social engagement or those who may be at risk in the future (Canadian Institute for Health Information, 2008). However, our findings suggest limits to such an approach; our data-driven machine-learning models predicted social engagement with only moderate predictive performance. This approach is further constrained by limited evidence regarding effective interventions to address social engagement in LTC home settings (Mikkelsen et al., 2019) and may ignore the underlying determinants of social isolation in LTC homes altogether (Theurer et al., 2015).

For research, these exploratory, hypothesis-generating findings have implications for future studies. The results may suggest that, in LTC homes, the widespread impacts of disability with accompanying loss of independence and social network coupled with the pervasive effects of ageism and ableism, and the lack of sex differences may be an indication that gender, as a “power relation” (Morgan et al., 2016), may be flattened; in this setting, irrespective of gender, residents have typically shed previous occupational and household roles, rules are set for them by the LTC home, and decision-making is often shared with or assumed by others.

This study used a large population-based sample of residents of LTC homes and detailed clinical assessment data, including over 600 variables, and covering a 10-year period. To our knowledge, this study is among the initial efforts to build predictive models for the ISE among residents of LTC homes and evaluate their performance in sex-stratified analysis to explore potential gender differences. This study, however, does have some limitations. Firstly, the study was limited to one Canadian province, Ontario, so the findings may not be generalizable to other populations. Secondly, the analysis was based on the original version of the ISE (Mor et al., 1995). While the ISE is still widely used in research and reporting in LTC homes, after exploring the content validity and internal consistency of the ISE, two items were replaced in the Revised Index of Social Engagement (RISE) (Gerritsen et al., 2008). This highlights an ongoing challenge for research and reporting on the social connection in LTC settings whereby, without broad consensus on the best approaches to measurement, diverse and sometimes untested measures have been used. Thirdly, while the data are population-based and offer the necessarily large sample with which to conduct the analyses, they must still be considered in context with potential for random error and information bias which applies irrespective of the analytical approach; issues including data collection burdens imposed on staff and residents as well as the emphasis on medical care have been described for the RAI-MDS (Armstrong et al., 2017). Fourthly, as a secondary data analysis, these clinical assessment data lack important information on individual-level predictors of social engagement, for example, measures of personality which have been shown to predict other aspects of social connection (Altschul et al., 2021), as well potentially important home-level variables, such as staffing, care philosophy, or the built environment of LTC homes. Future studies would benefit from linking external datasets or primary data collection to describe characteristics of the LTC home and surrounding area (Clemens et al., 2022).

## Conclusion

In this study, the important clinical assessment variables for predicting social engagement among residents of LTC homes were identified and used to build predictive machine-learning models for the ISE. The findings imply few sex differences in the predictors of social engagement in this setting. Sex-specific predictive models only marginally outperformed general non-sex-specific models which were built on both sexes. Predictive models based on the ISE could be used to identify residents who are at risk of low social engagement for targeted interventions; however, the current models suggested only moderate performance and there is limited evidence on effective interventions in this context. While these findings are useful for hypothesis generation and future research on social connection in LTC homes, expanded data collection and improved data quality are necessary for these models to assess potential gender differences or be used in planning and providing care related to social engagement in LTC homes.

## Supplemental Material

Supplemental Material - Prediction of Social Engagement in Long-Term Care Homes by Sex: A Population-Based Analysis Using Machine Learning Supplemental Material for Prediction of Social Engagement in Long-Term Care Homes by Sex: A Population-Bases Analysis Using Machine by Ali Abedi, Shehroz S. Khan, Andrea Iaboni, Susan E. Bronskill, and Jennifer Bethell in Journal of Applied Gerontology.

## Data Availability

The dataset from this study is held securely in coded form at ICES. While legal data sharing agreements between ICES and data providers (e.g., healthcare organizations and government) prohibit ICES from making the dataset publicly available, access may be granted to those who meet pre-specified criteria for confidential access, available at email: das@ices.on.ca). The full dataset creation plan and underlying analytic code are available from the authors upon request, understanding that the computer programs may rely upon coding templates or macros that are unique to ICES and are therefore either inaccessible or may require modification.
