# Supplementary material for: Prediction of Social Engagement in Long-Term Care Homes by Sex: A Population-Based Analysis Using Machine Learning
Source: J Appl Gerontol. 2024 Oct 12;44(6):902–15. doi: 10.1177/07334648241290589 (PMC12059231; doi:10.1177/07334648241290589)
Supplement: Supplemental Material - Prediction of Social Engagement in Long-Term Care Homes by Sex: A Population-Based Analysis Using Machine Learning [file sj-pdf-1-jag-10.1177_07334648241290589.pdf]

Table S1. The list of predictor variables used for the prediction of the Index of Social Engagement (ISE) in the study and their coding.

#	Variable Name*	Variable Category*	Coding*
1	Average Time Involved in Activities	Activity Pursuit Patterns	0 = Most: More Than 2/3 of Time 1 = Some: from 1/3 to 2/3 of Time 2 = Little: Less Than 1/3 of Time 3 = None 8 = Comatose
2	Prefer Trips or Shopping	Activity Pursuit Patterns	0 = No 1 = Yes 8 = Comatose
3	Prefer Talking or Conversing	Activity Pursuit Patterns	0 = No 1 = Yes 8 = Comatose
4	Prefer Playing Crafts/arts	Activity Pursuit Patterns	0 = No 1 = Yes 8 = Comatose
5	Prefer Helping Others	Activity Pursuit Patterns	0 = No 1 = Yes 8 = Comatose
6	Prefer Reading/writing	Activity Pursuit Patterns	0 = No 1 = Yes 8 = Comatose
7	Prefer Exercise/sports	Activity Pursuit Patterns	0 = No 1 = Yes 8 = Comatose
8	Outside Facility Activity Preference	Activity Pursuit Patterns	0 = No 1 = Yes 8 = Comatose
9	Inside Facility/off Unit Activity Preference	Activity Pursuit Patterns	0 = No 1 = Yes 8 = Comatose
10	Day or Activity Room Activity Preference	Activity Pursuit Patterns	0 = No 1 = Yes 8 = Comatose
11	Watching TV	Activity Pursuit Patterns	0 = No 1 = Yes 8 = Comatose
12	Spiritual/religious Activities	Activity Pursuit Patterns	0 = No 1 = Yes 8 = Comatose
13	Walk/wheeling Outdoors	Activity Pursuit Patterns	0 = No 1 = Yes 8 = Comatose
14	Own Room	Activity Pursuit Patterns	0 = No 1 = Yes 8 = Comatose
15	Evening Time Awake	Activity Pursuit Patterns	0 = No 1 = Yes 8 = Comatose
16	Afternoon Time Awake	Activity Pursuit Patterns	0 = No 1 = Yes 8 = Comatose
17	Prefer Gardening or Plants	Activity Pursuit Patterns	0 = No 1 = Yes 8 = Comatose
18	Prefer Music	Activity Pursuit Patterns	0 = No 1 = Yes 8 = Comatose
19	Resists Care	Behaviour Symptoms	0 = Behaviour Not Exhibited in Last Seven (7) Days 1 = Behaviour of This Type Occurred One (1) to Three (3) Days in Last Seven (7) Days 2 = Behaviour of This Type Occurred Four (4) to Six (6) Days in Last Seven (7) Days, but Less Than Daily 3 = Behaviour of This Type Occurred Daily in Last Seven (7) Days 8 = Comatose
20	Able to Locate Own Room	Cognitive Patterns	0 = Ok 1 = Problem 8 = Comatose
21	Able to Determine That is in a Facility	Cognitive Patterns	0 = No 1 = Yes 8 = Comatose

22	Able to Identify the Current Season	Cognitive Patterns	0 = No 1 = Yes 8 = Comatose
23	Able to Distinguish Staff Names/faces	Cognitive Patterns	0 = No 1 = Yes 8 = Comatose
24	Long-term Memory Problem	Cognitive Patterns	0 = No 1 = Yes 8 = Comatose
25	Cognitive Skills for Daily Decision Making	Cognitive Patterns	0 = Independent 1 = Modified Independence 2 = Moderately Impaired 3 = Severely Impaired 8 = Comatose
26	Able to Recall Staff Names/faces	Cognitive Patterns	0 = Ok 1 = Problem 8 = Comatose
27	Periods of Restlessness	Cognitive Patterns	0 = Behaviour Not Present 1 = Behaviour Present, Not of Recent Onset 2 = Behaviour Present, Over Last Seven (7) Days Appears Different from Resident's Usual Functioning, E.g. New Onset or Worsening 8 = Comatose
28	Easily Distracted	Cognitive Patterns	0 = Behaviour Not Present 1 = Behaviour Present, Not of Recent Onset 2 = Behaviour Present, Over Last Seven (7) Days Appears Different from Resident's Usual Functioning, E.g. New Onset or Worsening 8 = Comatose
29	Episodes of Disorganized Speech	Cognitive Patterns	0 = Behaviour Not Present 1 = Behaviour Present, Not of Recent Onset 2 = Behaviour Present, Over Last Seven (7) Days Appears Different from Resident's Usual Functioning, E.g. New Onset or Worsening 8 = Comatose
30	Mental Function Varies Over the Course of the Day	Cognitive Patterns	0 = Behaviour Not Present 1 = Behaviour Present, Not of Recent Onset 2 = Behaviour Present, Over Last Seven (7) Days Appears Different from Resident's Usual Functioning, E.g. New Onset or Worsening 8 = Comatose
31	Periods of Lethargy	Cognitive Patterns	0 = Behaviour Not Present 1 = Behaviour Present, Not of Recent Onset 2 = Behaviour Present, Over Last Seven (7) Days Appears Different from Resident's Usual Functioning, E.g. New Onset or Worsening 8 = Comatose

32	Signs or Gestures or Sounds	Communication/ Hearing Patterns	0 = No 1 = Yes 8 = Comatose
33	Speech Clarity	Communication/hearing Patterns	0 = Clear Speech Distinct, Intelligible Words 1 = Unclear Speech Slurred, Mumbled Words 2 = No Speech Absence of Spoken Words 8 = Comatose
34	Making Self Understood	Communication/hearing Patterns	0 = Understood 1 = Usually Understood, Difficulty Finding Words or Finishing Thoughts 2 = Sometimes Understood, Ability is Limited to Making Concrete Requests 3 = Rarely/never Understood 8 = Comatose
35	Understands Others	Communication/hearing Patterns	1 = Usually Understands 2 = Sometimes Understands
36	Bowel Incontinence	Continence	0 = Continent- Complete Control 1 = Usually Continent- Incontinent Less Than Weekly 2 = Occasionally Incontinent- Incontinent Once a Week 3 = Frequently Incontinent- Incontinent 2 to 3 Times a Week 4 = Incontinent- Incontinent All (or Almost All) of the Time
37	Bladder Incontinence	Continence	0 = Continent- Complete Control (includes Use of Indwelling Urinary Catheter That Does Not Leak Urine) 1 = Usually Continent- Incontinent Episodes Once a Week or Less 2 = Occasionally Incontinent- Incontinent 2+ Times a Week but Not Daily 3 = Frequently Incontinent- Tended to Be Incontinent Daily, but Some Control Present (e.g. on Day Shift) 4 = Incontinent- Had Inadequate Control with Multiple Daily Episodes
38	Dementia Other Than Alzheimer’s Disease	Disease Diagnoses	0 = No 1 = Yes 8 = Comatose
39	Unsteady Gait	Health Conditions	0 = No 1 = Yes 8 = Comatose
40	Number of Medications (per Week)	Medications	Continuous 0 — 99
41	Antipsychotic (per Week)	Medications	Continuous 0 — 7
42	Diuretic	Medications	Continuous 0 — 7
43	Withdrawal from Activities of Interest	Mood and Behaviour Patterns	0 = Indicator Not Exhibited in Last 30 Days 1 = Indicator of This Type Exhibited Up to 5 Days a Week 2 = Indicator of This Type Exhibited Daily or Almost Daily

			(6, 7 Days a Week) 8 = Comatose
44	Mood Persistence	Mood and Behaviour Patterns	0 = No Mood Indicators Observed 1 = Indicators Present, Easily Altered 2 = Indicators Present, Not Easily Altered 8 = Comatose
45	Sad, Pained, Worried Facial Expressions	Mood and Behaviour Patterns	0 = Indicator Not Exhibited in Last 30 Days 1 = Indicator of This Type Exhibited Up to 5 Days a Week 2 = Indicator of This Type Exhibited Daily or Almost Daily (6, 7 Days a Week) 8 = Comatose
46	Repetitive Physical Movements	Mood and Behaviour Patterns	0 = Indicator Not Exhibited in Last 30 Days 1 = Indicator of This Type Exhibited Up to 5 Days a Week 2 = Indicator of This Type Exhibited Daily or Almost Daily (6, 7 Days a Week) 8 = Comatose
47	Reduced Social Interaction	Mood and Behaviour Patterns	0 = Indicator Not Exhibited in Last 30 Days 1 = Indicator of This Type Exhibited Up to 5 Days a Week 2 = Indicator of This Type Exhibited Daily or Almost Daily (6, 7 Days a Week) 8 = Comatose
48	Physically Abusive Behavioral Symptoms	Mood and Behaviour Patterns	0 = Behaviour Not Exhibited in Last Seven (7) Days 1 = Behaviour of This Type Occurred One (1) to Three (3) Days in Last Seven (7) Days 2 = Behaviour of This Type Occurred Four (4) to Six (6) Days in Last Seven (7) Days, but Less Than Daily 3 = Behaviour of This Type Occurred Daily in Last Seven (7) Days 8 = Comatose
49	Unpleasant Mood in Morning	Mood and Behaviour Patterns	0 = Indicator Not Exhibited in Last 30 Days 1 = Indicator of This Type Exhibited Up to 5 Days a Week 2 = Indicator of This Type Exhibited Daily or Almost Daily (6, 7 Days a Week) 8 = Comatose
50	Has Dentures or Removable Bridge	Oral/dental Status	0 = No 1 = Yes 8 = Comatose
51	Mechanically Altered Diet	Oral/nutritional Status	0 = No 1 = Yes
52	Leaves 25% or More of Food Uneaten	Oral/nutritional Status	0 = No 1 = Yes 8 = Comatose
53	Dietary Supplement Between Meals	Oral/nutritional Status	0 = No 1 = Yes 8 = Comatose
54	Cognitive Performance Scale	Outcome Scale	0–6 Higher Scores Indicate More Severe Cognitive Impairment. Five Cognitive Performance Scale Items

			Comatose Short-term Memory Cognition Skills for Daily Decision-making Expressive Communication Eating
55	Pressure Ulcer Risk Scale	Outcome Scale	0–8 Higher Scores Indicate a Higher Relative Risk for Developing a Pressure Ulcer. Seven Interrai Pressure Ulcer Risk Scale Items Bed Mobility Self-performance Walk in Room Self-performance Bowel Incontinence Shortness of Breath Daily Pain Weight Loss History of Resolved Ulcer or Pressure Ulcer
56	Activities of Daily Living (ADL) - Long Form	Outcome Scale	0–28 Higher Scores Indicate More Impairment of Self-sufficiency in ADL Performance. Seven ADL Long Form Items Mobility in Bed Transfers Locomotion Dressing Eating Toilet Use Personal Hygiene
57	Aggressive Behaviour Scale	Outcome Scale	0–12 Higher Scores Indicate Higher Levels of Aggressive Behaviour. Four Aggressive Behaviour Scale Items Verbally Abusive Physically Abusive Socially Inappropriate/disruptive Behaviour Resists Care
58	Activities of Daily Living (ADL) - Short Form	Outcome Scale	0–16 Higher Scores Indicate More Impairment of Self-sufficiency in ADL Performance. Four ADL Short Form Items Personal Hygiene Toilet Use Locomotion Eating
59	Bed Mobility	Physical Functioning and Structural Problems	0 = Independent 1 = Supervision 2 = Limited Assistance 3 = Extensive Assistance 4 = Total Dependence 8 = Activity Did Not Occur During Entire 7 Days
60	Toilet Use	Physical Functioning and Structural Problems	0 = Independent 1 = Supervision 2 = Limited Assistance 3 = Extensive Assistance 4 = Total Dependence 8 = Activity Did Not Occur During Entire 7 Days
61	Transfer Between Surfaces	Physical Functioning and Structural Problems	0 = Independent 1 = Supervision 2 = Limited Assistance 3 = Extensive Assistance 4 = Total Dependence 8 = Activity Did Not Occur During Entire 7 Days
62	Bed Rails Used for Bed Mobility or Transfer	Physical Functioning and Structural Problems	0 = No 1 = Yes 8 = Comatose
63	Task Segmentation	Physical Functioning and Structural Problems	0 = No 1 = Yes 8 = Comatose

64	Locomotion on Unit	Physical Functioning and Structural Problems	0 = No Set-up or Physical Help from Staff 1 = Set-up Help Only 2 = One Person Physical Assist 3 = Two + Persons Physical Assist 8 = Activity Did Not Occur During Entire 7 Days
65	Arm Voluntary Movement	Physical Functioning and Structural Problems	0 = No Loss 1 = Partial Loss 2 = Full Loss
66	Walk in Corridor	Physical Functioning and Structural Problems	0 = Independent. 1 = Supervision. 2 = Limited Assistance. 3 = Extensive Assistance. 4 = Total Dependence. 8 = Activity Did Not Occur During Entire 7 Days
67	Independent Eating	Physical Functioning and Structural Problems	0 = Independent 1 = Supervision 2 = Limited Assistance. 3 = Extensive Assistance. 4 = Total Dependence. 8 = Activity Did Not Occur During Entire 7 Days
68	Balance Problem While Sitting	Physical Functioning and Structural Problems	0 = Maintained Position as Required During Test 1 = Unsteady, but Able to Rebalance Self Without Physical Support 2 = Partial Physical Support During Test; or Stands but Does Not Follow Directions for Test 3 = Not Able to Attempt Test Without Physical Help
69	Locomotion Off Unit	Physical Functioning and Structural Problems	0 = Independent 1 = Supervision 2 = Limited Assistance 3 = Extensive Assistance 4 = Total Dependence 8 = Activity Did Not Occur During Entire 7 Days
70	Bathing Self-performance	Physical Functioning and Structural Problems	0 = Independent: No Help Provided 1 = Supervision: Oversight Help Only 2 = Physical Help Limited to Transfer Only 3 = Physical Help in Part of Bathing Only 4 = Total Dependence 8 = Bathing Did Not Occur During Entire Seven (7) Days
71	Balance Problem While Standing	Physical Functioning and Structural Problems	0 = Maintained Position as Required During Test 1 = Unsteady, but Able to Rebalance Self Without Physical Support 2 = Partial Physical Support During Test; or Stands but Does Not Follow Directions for Test 3 = Not Able to Attempt Test Without Physical Help
72	Independent Dressing	Physical Functioning and	0 = Independent 1 = Supervision 2 = Limited Assistance.

		Structural Problems	3 = Extensive Assistance. 4 = Total Dependence. 8 = Activity Did Not Occur During Entire 7 Days
73	Neck Range of Motion	Physical Functioning and Structural Problems	0 = No Limitation 1 = Limitation on One Side 2 = Limitation on Both Sides
74	Arm Range of Motion	Physical Functioning and Structural Problems	0 = No Limitation 1 = Limitation on One Side 2 = Limitation on Both Sides
75	Leg Range of Motion	Physical Functioning and Structural Problems	0 = No Limitation 1 = Limitation on One Side 2 = Limitation on Both Sides
76	Personal Hygiene	Physical Functioning and Structural Problems	0 = Independent 1 = Supervision 2 = Limited Assistance. 3 = Extensive Assistance. 4 = Total Dependence. 8 = Activity Did Not Occur During Entire 7 Days
77	Bathing Support	Physical Functioning and Structural Problems	0 = No Set-up or Physical Help from Staff 1 = Set-up Help Only 2 = One Person Physical Assist 3 = Two + Persons Physical Assist 8 = Bathing Did Not Occur During Entire Seven (7) Days
78	Foot Range of Motion	Physical Functioning and Structural Problems	0 = No Limitation 1 = Limitation on One Side 2 = Limitation on Both Sides
79	Prefer Playing Cards and Other Games	Prefer Playing Cards and Other Games	0 = No 1 = Yes 8 = Comatose
80	Strong Identification with Past Roles and Life Status	Psychosocial Well-being	0 = No 1 = Yes 8 = Comatose
81	Perceiving the Difference of Daily Life	Psychosocial Well-being	0 = No 1 = Yes 8 = Comatose
82	Vision	Vision Patterns	0 = Adequate Sees Fine Detail, Including Regular Print in Newspapers/books 1 = Impaired Sees Large Print, but Not Regular Print in Newspapers/books 2 = Moderately Impaired Limited Vision; Not Able to See Newspaper Headlines, but Can Identify Objects 3 = Highly Impaired Object Identification in Question, but Eyes Appear to Follow Objects 4 = Severely Impaired No Vision or Sees Only Light, Colors, or Shapes; Eyes Do Not Appear to Follow Objects 8 = Comatose
83	Visual Appliances	Vision Patterns	0 = No 1 = Yes 8 = Comatose

[*] The name, category, and coding of the variables were taken from the RAI-MDS guidelines for full annual assessments (Canadian Institute for Health Information, 2010)
